# Coupling of Co-expression Network Analysis and Machine Learning Validation Unearthed Potential Key Genes Involved in Rheumatoid Arthritis

**DOI:** 10.3389/fgene.2021.604714

**Published:** 2021-02-11

**Authors:** Jianwei Xiao, Rongsheng Wang, Xu Cai, Zhizhong Ye

**Affiliations:** ^1^Department of Rheumatology and Immunology, Shenzhen Futian Hospital for Rheumatic Diseases, Shenzhen, China; ^2^Department of Rheumatology, Shanghai Guanghua Hospital of Integrated Traditional Chinese and Western Medicine, Shanghai, China

**Keywords:** rheumatoid arthritis, artificial intelligence, machine learning, WGCNA, diagnosis, prognosis

## Abstract

Rheumatoid arthritis (RA) is an incurable disease that afflicts 0.5–1.0% of the global population though it is less threatening at its early stage. Therefore, improved diagnostic efficiency and prognostic outcome are critical for confronting RA. Although machine learning is considered a promising technique in clinical research, its potential in verifying the biological significance of gene was not fully exploited. The performance of a machine learning model depends greatly on the features used for model training; therefore, the effectiveness of prediction might reflect the quality of input features. In the present study, we used weighted gene co-expression network analysis (WGCNA) in conjunction with differentially expressed gene (DEG) analysis to select the key genes that were highly associated with RA phenotypes based on multiple microarray datasets of RA blood samples, after which they were used as features in machine learning model validation. A total of six machine learning models were used to validate the biological significance of the key genes based on gene expression, among which five models achieved good performances [area under curve (AUC) >0.85], suggesting that our currently identified key genes are biologically significant and highly representative of genes involved in RA. Combined with other biological interpretations including Gene Ontology (GO) analysis, protein–protein interaction (PPI) network analysis, as well as inference of immune cell composition, our current study might shed a light on the in-depth study of RA diagnosis and prognosis.

## Introduction

Rheumatoid arthritis (RA) is a long-term autoimmune disease that provokes synovial inflammation (Song and Lin, 2017) and predominantly inflicts accumulative damage on joints (Smolen et al., 2016). Epidemiology studies revealed ~49,000 RA-associated deaths globally in 2010 (Lozano et al., 2012). The incidence of RA was higher in females than in males, and its pathogenesis was mainly dependent on genetic factors, especially immune-associated genes (Song and Lin, 2017). Due to the fact that RA could eventually result in varying degrees of joint impairments or even disability and severely affect the life quality of the victims, it has imposed a heavy burden on the society (Lundkvist et al., 2008). Regrettably, RA is still an incurable disease; therefore, timely intervention will be necessary. Aside from acute RA onset that immediately perturbs the immune system, most pre-clinical RA, whose clinical symptoms are still inconspicuous, could be abrogated through customized interventions, and the resulting establishment of RA could also be prevented (Smolen et al., 2016). As noted above, accurate diagnosis and improved prognosis are crucial for monitoring RA, although the heterogeneity of RA could undermine the effectiveness of these approaches. The heterogeneity of RA is characterized by its clinical symptoms and pathogenesis that vary across patients who receive the same diagnosis (Smolen et al., 2016). Thus, there is a fundamental need for understanding the molecular mechanism underlying the heterogeneous RA to improve both diagnostic and prognostic outcomes.

Microarray technology is a widely used, high-throughput and robust approach that allows the simultaneous detection of gene expression profile of thousands of genes. Considerable information provided by microarray has been deposited in Gene Expression Omnibus (GEO), through which public data could be integrated and re-analyzed, providing valuable information and novel perspectives regarding different diseases. Thanks to the development of bioinformatics, numerous analytical methods have been implemented for data mining from public databases, among which weighted gene co-expression network analysis (WGCNA) is a well-known approach for gene co-expression analysis. WGCNA was initially proposed by scholars Zhang and Horvath (2005), the corresponding concepts were later integrated into the R package, which can be used in various aspects, such as establishment of a weighted gene co-expression network, detection of highly correlated gene modules, association of gene modules with clinical traits, and identification of intra-modular hub genes (Langfelder and Horvath, 2008). WGCNA is widely used in bioinformatics analysis of many diseases, including RA. For instance, Gene Ontology (GO) enrichment of modules defined by WGCNA showed several immune response-related genes that play potential roles in RA (Ma et al., 2017). In addition, pivotal differentially expressed genes (DEGs) associated with RA and corresponding networks have been developed by combined utilization of DEG analysis and WGCNA (He et al., 2019). Moreover, WGCNA followed by *in vivo* validation showed that imatinib could mitigate the inflammatory responses in RA through suppressing CSF1R (Hu et al., 2019).

As a subset of artificial intelligence, machine learning has emerged as a valuable tool in the field of biology, for instance, Le et al. (2017) developed a web-based tool ET-CNN, which was specialized for distinguishing electron transport protein through combined utilization of convolutional neural network (CNN) and position-specific scoring matrices (PSSM); similarly, coupling of deep gated recurrent units and PSSM profiles was used to identify fertility-associated proteins (Le, 2019), whereas deep-neural network-based natural language processing was used to decipher S-sulfenylation sites from protein sequences (Do et al., 2020). In the clinical setting, such technology also bears the potential of disease detection and classification (Cruz and Wishart, 2007), but it should be noted that the prediction accuracy of machine learning model is often inversely correlated with the number of features (variables), that careful consideration should be given when selecting limited number of appropriate features, and that these parameters should be as informative as possible (Jamshidi et al., 2019). Although machine learning has been widely used to predict arthritis based on different features, such as immunological profile (Van Nieuwenhove et al., 2019), as well as integration of clinical, therapeutic, and laboratory information (Ceccarelli et al., 2018), implementation of biomarker-based machine learning model validation in RA is relatively limited. Moreover, the effectiveness of prediction/classification of machine learning models could reflect the quality of input features (Jamshidi et al., 2019). Therefore, the present study aims at employing machine learning model validation to further verify the biological significance of biomarkers determined by WGCNA.

Specifically, the current study was designed in conformity with the following major points to achieve the intended goal: first, we defined the intersection between hub genes in the training set (determined by WGCNA) and DEGs in the test set as key genes and verified the biological significance of these markers in a tissue sample dataset. Second, the key genes were used as features in machine learning model validation; the key genes were used as features in six different machine learning classification models, the performances of these models were further evaluated, and the biological significance of the key genes was further confirmed.

## Materials and Methods

### Data Collection

Gene expression data were downloaded from the GEO database (http://www.ncbi.nlm.nih.gov/geo/) on July 15, 2020; we screened GEO microarray datasets in compliance with the following criteria: (1) samples of RA patients and healthy controls (HCs) were collected before receiving pharmacotherapy or other treatments that might affect gene expression profiles; (2) HCs are not necessarily the control used in RA research, as long as these samples represent the gene expression in healthy population. After careful consideration, a total of 12 GEO datasets (Table 1) were applied in this study, including the training set composed of 11 profiles (GSE93272, GSE45291, GSE74143, GSE65010, GSE15573, GSE61635, GSE65391, GSE138458, GSE143272, GSE113469, GSE50772) containing blood samples from 419 RA patients and 318 HCs, along with the test set based on one dataset (GSE55457) containing tissue samples of 10 RA patients and 13 HCs. Expression matrices of the 12 GEO datasets are available in Supplementary Tables 1.1, 1.2.

**Table 1:** Information of 12 GEO datasets.

**No. of GEO profile**	**Batch number**	**Experiment type**	**Source**	**Case**	**Control**	**Platform**	**Category**
GSE93272	B1	Expression profiling by array	Whole blood	0	43	GPL570	Training set
GSE45291	B2	Expression profiling by array	Whole blood	0	20	GPL13158	Training set
GSE74143	B3	Expression profiling by array	Whole blood	377	0	GPL13158	Training set
GSE65010	B4	Expression profiling by array	Peripheral blood	24	24	GPL570	Training set
GSE15573	B5	Expression profiling by array	Peripheral blood	18	15	GPL6102	Training set
GSE61635	B6	Expression profiling by array	Whole blood	0	30	GPL570	Training set
GSE65391	B7	Expression profiling by array	Whole blood	0	72	GPL10588	Training set
GSE138458	B8	Expression profiling by array	Plasma	0	23	GPL10558	Training set
GSE143272	B9	Expression profiling by array	Peripheral blood	0	51	GPL10558	Training set
GSE113469	B10	Expression profiling by array	Peripheral blood	0	20	GPL10558	Training set
GSE50772	B11	Expression profiling by array	Peripheral blood	0	20	GPL570	Training set
GSE55457	B12	Expression profiling by array	Frozen tissue	13	10	GPL96	Testing set

*The training set comprised 11 datasets derived from blood samples, whereas the test set was based on 1 tissue dataset*.

### Data Pre-processing

During the filtering process, intersection of the identified gene symbols across all gene expression arrays (which were common to 12 datasets) was retained and used for integration of 11 training datasets. In order to eliminate potential batch effects that resulted from systematic and non-biological differences among the 11 batches in the training set, we used t-SNE dimensionality reduction algorithm to determine the existence of batch effects. Subsequently, the removeBatch Effect function in the R (R statistical software, version 3.6.3) package limma (version 3.42.2) (Ritchie et al., 2015) was used to eliminate the batch effects and preserve the differences between different clinical status (RA/control). Finally, Quantile method in voom function of the limma package was implemented for data normalization.

### DEG Analysis

The limma package was run to discover DEGs based on the criteria of |log_2_FC| >0.5 and *p* < 0.05, and the DEGs were displayed on a volcano plot. To further visualize the expression of DEGs in different groups, the top 40 DEGs were selected based on the p-values to generate a heatmap using the R package ComplexHeatmap (version 2.2.0) (Gu et al., 2016).

### WGCNA Analysis

The R package WGCNA (version 1.69) (Langfelder and Horvath, 2008) was used to perform WGCNA analysis in R. WGCNA parameters were default unless specified otherwise. Firstly, the correlation matrix between the genes was calculated using s_mn_ (co-expression similarity) = |cor(m, n)| based on the gene expression array, after which a weighted undirected network was established using a_mn_ (adjacency between gene m and gene n) = s_mn_^β^, the appropriate power parameter β = 8 was determined by pickSoftThreshold function (Langfelder and Horvath, 2008). Secondly, all genes were divided into different modules according to the dynamic mixed method of similarity. Next, the module-trait relationship was calculated according to the correlation between the gene modules and clinical information, through which two modules with the strongest positive or negative correlation with the sample status were screened out. Groups of genes in these respective two modules were named module genes, and the intersection of module genes and DEGs in the training set was named hub genes.

### Network Analysis of Module Genes/Hub Genes

Network analysis of the module genes at transcriptome level was performed using Cytoscape (version 3.6.1) (Shannon et al., 2003) in accordance with the following criteria: node score (weighted adjacency between two nodes) cut-off ≥0.42 and degree (number of edges possessed by a node) cut-off ≥50, whereby co-expression among module genes was intuitively visualized. As for interaction at protein level, a protein–protein interaction (PPI) network was constructed by using hub genes as queries against the STRING database (version 11.0) (Szklarczyk et al., 2017), and topological parameters of the network were analyzed locally using the Cytoscape plug-in “NetworkAnalyzer” (version 4.4.6). For in-depth analysis, the intersection between hub genes and DEGs in the test set was further defined as key genes.

### GO and KEGG Enrichment Analysis

The GO and Kyoto Encyclopedia of Genes and Genomes (KEGG) enrichment analysis of DEGs was implemented by the R package clusterProfiler (version 3.14.3) (Yu et al., 2012) and displayed in the dotplot, wherein enriched pathways were described using gene ratio, adjusted p-value, and count; enrichment with adjusted *p* < 0.1 was considered statistically significant. Enrichment analyses including GO and KEGG pathway analysis were performed each time when a new set of genes (DEGs, module genes, or hub genes) was defined.

### Inference of Immune Cells Composition in Tissue Samples

As RA could provoke inflammation (Song and Lin, 2017), it is reasonable that RA is accompanied by altered immune cells composition. After the validation of the key genes, we explored such alteration in RA tissue samples by comparing their normal counterparts. Briefly, deconvolution of cellular composition was carried out using CIBER-SORT algorithm (Cell-type Identification by Estimating Relative Subsets of Known RNA Transcripts) (Newman et al., 2015), a machine learning algorithm trained on 22 pre-defined distinct immune cell profiles corresponding to the expression patterns of 547 genes, whereby different immune cell compositions could be distinguished by these “molecular barcodes,” under a null hypothesis that none of the pre-defined cells were predicted to reside in a given sample (Newman et al., 2015). The analysis was carried out in compliance with a previously described protocol (Chen et al., 2018a), where 100 permutations were performed to ensure the statistical rigor. The resultant relative fractions of different immune cells were visualized using stacked barplot showing the percentages of immune cell components, and the significance of alterations was calculated using Wilcoxon rank-sum test (RA group vs. HC group) followed by p-value adjustment of multiple testing (q-value) and displayed on a volcano plot.

### Validation of the Key Genes and Their Biological Significance

To further confirm the biological significance of our currently identified key genes, machine learning models were employed. Initially, LASSO (least absolute shrinkage and selection operator) algorithm was performed on the key genes to select the modeling feature. LASSO was implemented by glmnet package (version 4.0.2) (Friedman et al., 2010) in R. Then, six machine learning classification models were used to train the predictive model. The training goal was to fit the appropriate model weights to predict whether the testing sample belongs to the RA sample or HC sample. The greedy algorithm was performed to optimize the hyperparameters by investigating all possible combination of hyperparameters within a certain range, and a 10-fold cross-validation was used to evaluate the availability of different combinations. The main parameters of the six models were as follows: (a) LASSO was equivalent to the logistic regression using L1 regularization optimization, the penalty coefficient C was set to 0.14, and the optimization algorithm was the “liblinear” method; (b) the penalty coefficient C in the support vector machine (SVM) model was set to 0.1, and the kernel function was set to radial basis function (RBF); (c) random forest (RF) stipulates that the maximum number of sub-decision trees was 200, and each sub-tree contained up to five features; (d) the maximum depth of eXtreme Gradient Boosting (Xgboost) was set to 4, the penalty coefficient λ was set to 10, and the learning rate was set to 0.001; (e) the back propagation neural network (BPNN) model was set to a single hidden layer network. The number of neurons in the hidden layer and the input layer remained the same. The activation function from the input layer to the hidden layer was set to the Relu function, and the value of the output layer compressed by the Sigmoid function was regarded as the probability value for output. The batch gradient descent method was used as the training method, the loss function was set to cross entropy function, the number of iterations was set to 1,000, and the learning rate was set to 0.001. (f) The CNN model, a 1D CNN model, contained two convolutional layers (conv1, conv2), two pooling layers (pool1, pool2), and two fully connected layers (fc1, fc2). Conv1 contained two output channels, its kernel size was set to 3, the stride was set to 1, and the padding was set to 1. Conv2 contained four output channels, its kernel size was set to 5, the stride was set to 1, and the padding was set to 0. The data underwent batch normalization after passing through each convolutional layer and then activated by the Relu function. Both the pool1 and pool2 used the max pooling as the pooling method, and the kernel size and stride were set to 2. After passing through the pool2 layer, the data were flattened and regarded as the input of the fc1 layer. The fc1 layer contained 10 neurons and used Relu as the activation function. The output of the fc2 layer was the probability value compressed by the Sigmoid function. Like BPNN, the batch gradient descent method was used as the training method, the loss function was set to cross entropy function, the number of iterations was set to 1,000, and the learning rate was set to 0.001. Each model was subjected to 1,000 times Monte Carlo cross-validations on the test set, which means that in each validation process, the training set was randomly partitioned in a 1/4 ratio for validation set and modeling set, respectively, whereas the test set remained unchanged during the process. The modeling set was used to fit the predictive model to predict the validation set or the test set. As a result, 1,000 values of six indicators [sensitivity, specificity, accuracy, positive predictive value (PPV), negative predictive value (NPV), area under curve (AUC)] were obtained, and the average value was used to evaluate the generalization performance of different models. The receiver operating characteristic (ROC) curve was drawn based on sensitivity [true positive rate (TPR)] and 1 – specificity [false positive rate (FPR)]. The area enclosed by the ROC curve and the x axis and y axis was called the AUC value, which was associated with the model performance. LASSO, SVM, and RF models were all integrated in the scikit-learn package (version 0.22.1) (Pedregosa et al., 2011), Xgboost was implemented by the xgboost package (version 1.1.0) (Chen and Guestrin, 2016), and the network structure of BPNN and CNN was built in the Pytorch framework (version 1.3.1) (Paszke et al., 2019). All models were implemented in python (version 3.7.6).

## Results

### Identification of DEGs and Their Functional Enrichment Analysis

**Figure 1:**
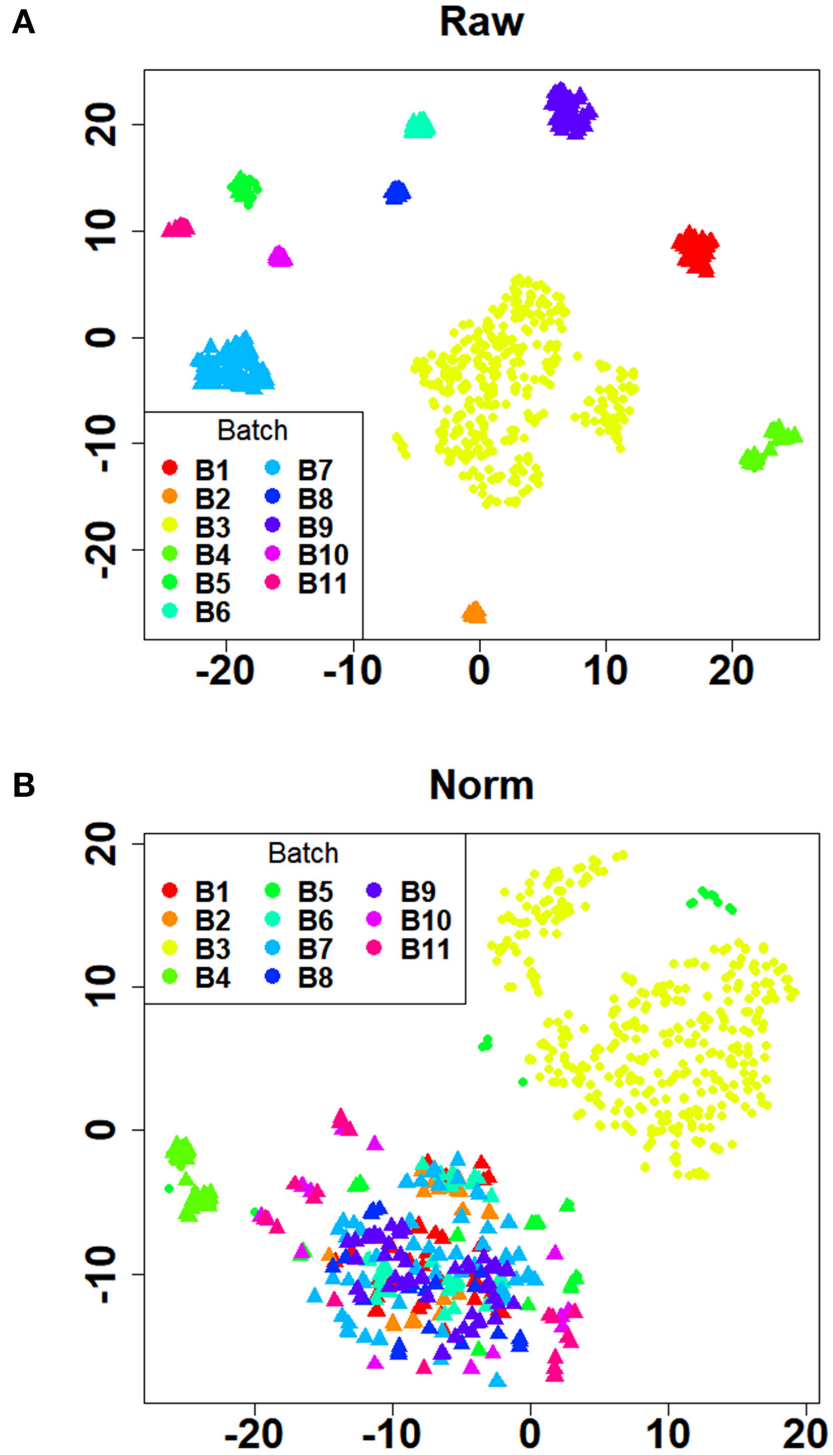
Verification of batch effect removal using t-SNE plots. **(A)** The distribution of the raw data after dimensionality reduction. **(B)** The distribution of the batch effect corrected data after dimensionality reduction. The dots with different colors represented samples in different batches. Healthy controls and rheumatoid arthritis samples were denoted by triangles and circles, respectively.

**Figure 2:**
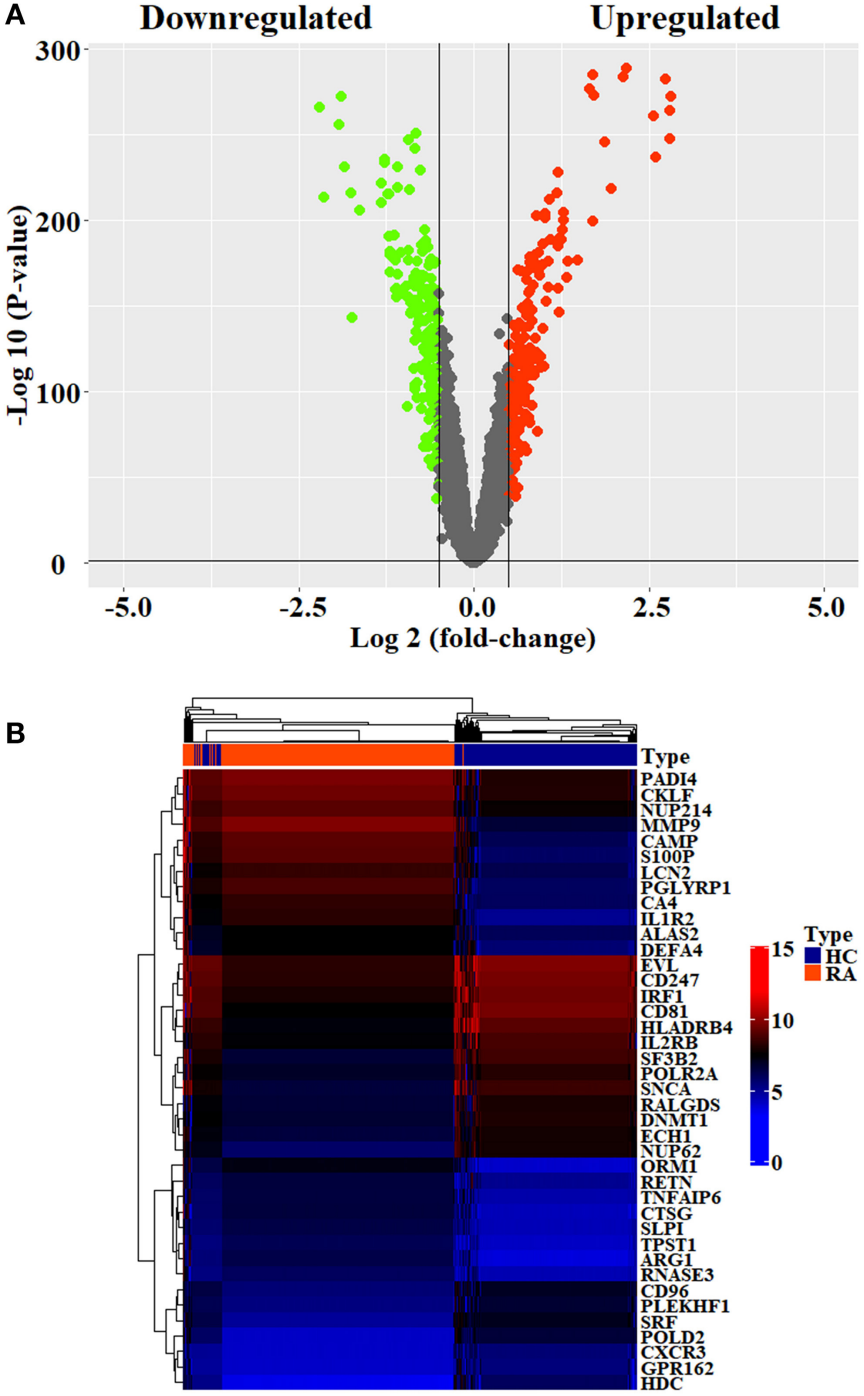
Differential analysis of datasets. **(A)** Volcano plots showing the differentially expressed genes screened by the criteria of |log2FC| >1 and *p* < 0.05. The upregulated and downregulated genes were denoted by red spots and green spots, respectively. **(B)** The top 10 DEGs with the smallest p-values in the upregulated and downregulated clusters were taken out, respectively, to generate heatmap.

**Figure 3:**
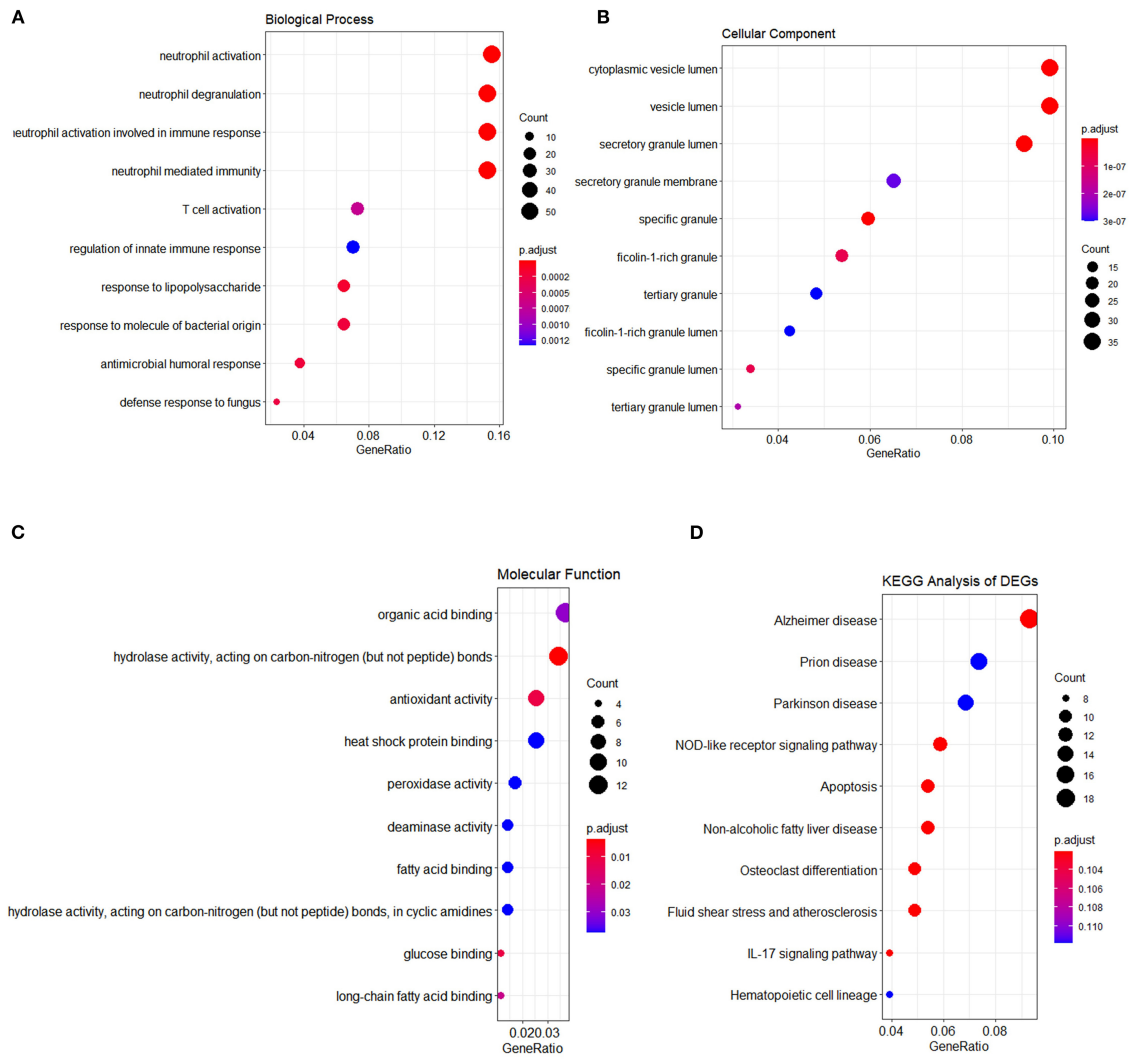
Functional enrichment analysis of DEGs. The results of three major GO enrichment categories are shown in **(A)** biological process, **(B)** cellular component, and **(C)** molecular function, respectively, along with **(D)** KEGG pathway. The dot size represented the count of differentially expressed genes, and the color depth represented the p-value-based significance.

Initially, 11 GEO datasets of blood sample were preprocessed in statistical analysis software R, whereby we found an obvious batch effect across different datasets (Figure 1A), which was subsequently corrected using removeBatchEffect in limma package (Figure 1B). Next, a total of 451 DEGs in the training set were discovered using limma package, among which 231 genes were upregulated, whereas 220 genes were downregulated. The volcano plot showing the upregulated and downregulated genes and non-DEGs was depicted in Figure 2A, whereas the top 40 DEGs ranked by p-values were used to generate the heatmap (Figure 2B). Detailed information concerning DEG analysis results was provided in Supplementary Table 2. Based on DEGs, GO term enrichment analysis was performed; our results were mainly focused on three different GO categories, namely, biological process (BP), molecular function (MF), and cellular component (CC), along with KEGG pathway analysis. Enrichment of DEGs was displayed in Figures 3A–D. With regard to BP, DEGs were significantly enriched in neutrophil activation, neutrophil degranulation, neutrophil activation involved in immune response, and neutrophil mediated immunity (Figure 3A). As for CC, DEGs were mainly implicated in cytoplasmic vesicle lumen, vesicle lumen, and secretory granule lumen (Figure 3B). With respect to MF, enrichment DEGs were found in the pathways concerning organic acid binding, antioxidant activity, and hydrolase activity acting on carbon–nitrogen (but not peptide) bonds and antioxidant activity (Figure 3C). In addition, significant enrichment of DEGs results in KEGG pathways of Alzheimer disease, NOD-like receptor signaling pathway, and apoptosis (Figure 3D). These significantly enriched pathways and terms improved our understanding of the regulatory roles of DEGs in RA occurrence and progression.

### Weighted Correlation Network Analysis

**Figure 4:**
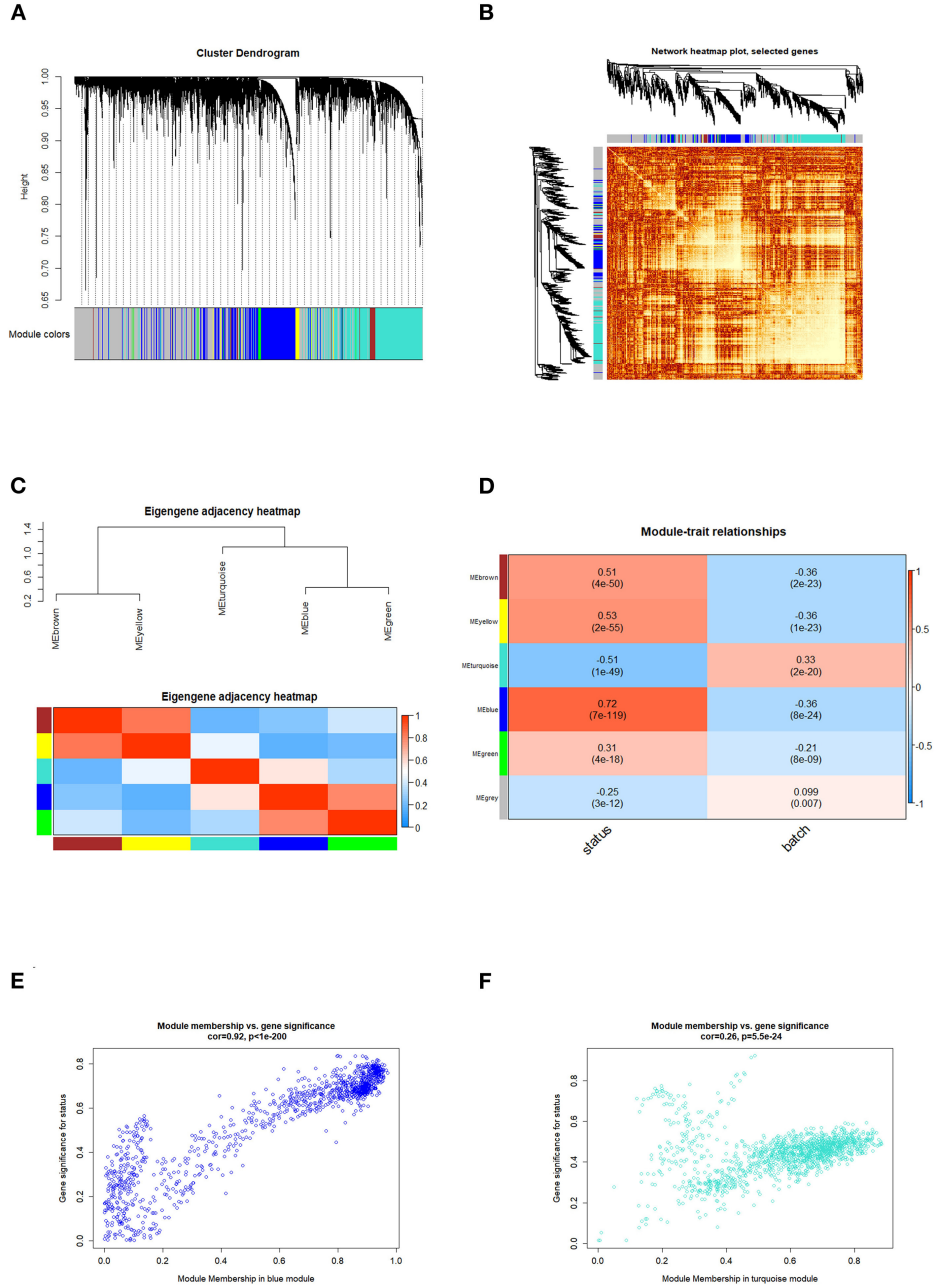
WGCNA analysis identified the gene modules related to RA. **(A)** The dendrogram showed that the molecules were classified into different gene modules based on the correlation analysis. Different colors represented the different modules, and the gray module represented genes that were unassigned to any network. **(B)** The network heatmap showed the correlation of genes in different modules, and the lighter areas represented the higher correlation. **(C)** Eigengene adjacency heatmap showed that aside from relatively strong correlation between the blue/green or yellow/brown modules, limited correlations were observed among other module pairs. **(D)** The heatmap of module trait relationships showed the correlation between each gene module and clinical status of samples. The red cube represented a positive correlation, whereas the blue cube represented a negative correlation. The consensus correlation between modules and phenotypes was reported as a number shown in each cube, with p value (in parenthesis) printed below the correlations. The blue/turquoise module had the most significant positive/negative correlation with the sample status, and the scatter plots (gene significance vs. module membership) of the blue and turquoise modules were shown in **(E,F)**, respectively.

**Figure 5:**
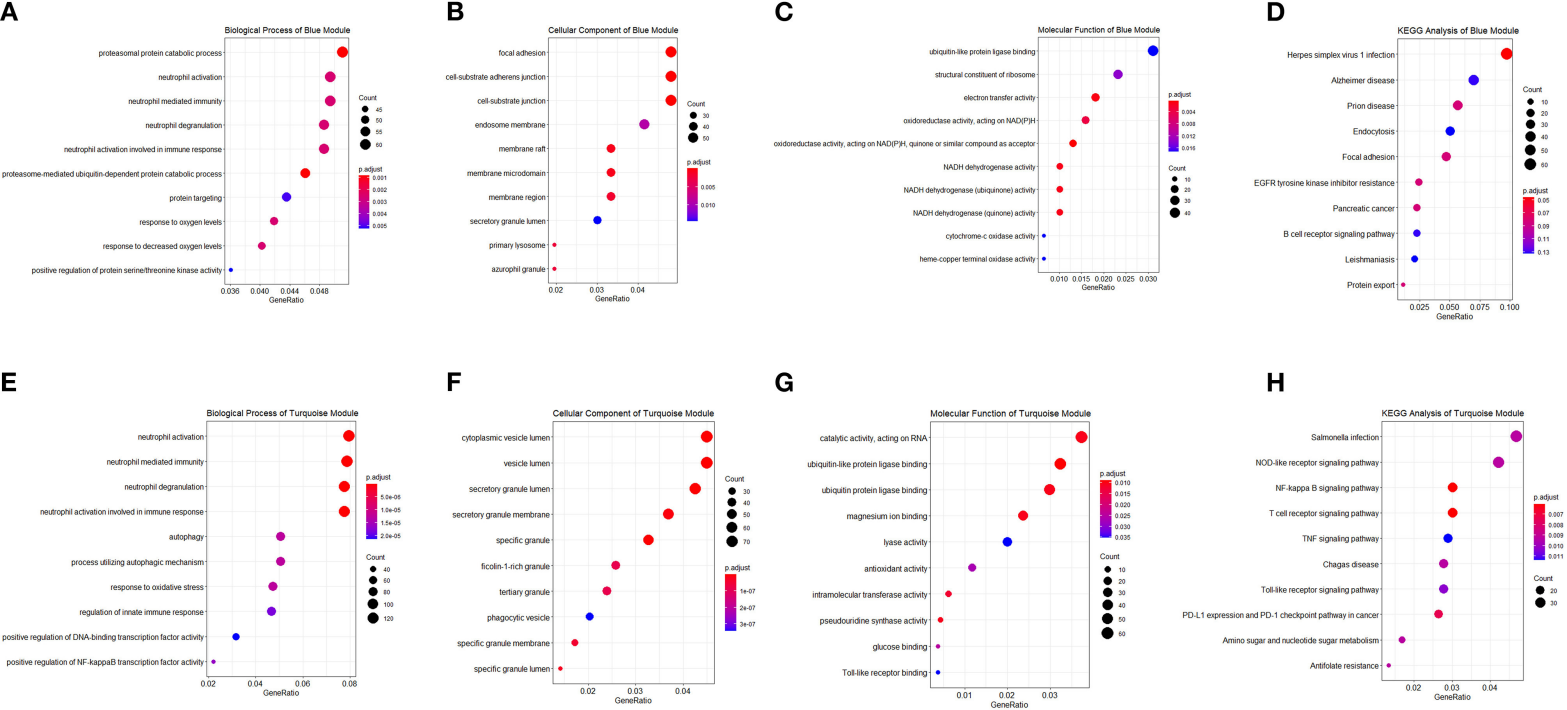
Functional enrichment analysis of module genes. Module genes in the blue and turquoise modules were analyzed, and enrichment categories of two modules are shown in **(A,E)** biological process, **(B,F)** cellular component, **(C,G)** molecular function, and **(D,H)** KEGG pathway, respectively. The dot size represented the count of differentially expressed genes, and the color depth represented the p-value-based significance.

Correlated genes usually exhibit identical or similar expression pattern. To gain a deeper insight into gene correlation in RA, we established a co-expression network to screen gene modules that contain highly correlated genes using WGCNA. As shown in Figure 4A, up to five modules (brown, yellow, turquoise, blue, and green) were mined, whereas the gray module represents genes without significant clustering information. The correlation of genes in different modules was shown in Figure 4B, whereas the eigengene adjacency heatmap and corresponding dendrogram (Figure 4C) showed the adjacencies among different modules, and the adjacencies between yellow/brown, blue/green, and blue/turquoise were relatively high, suggesting a positive intra-correlation within these module pairs (Figure 4D). Further analysis showed that the blue gene module had the most significant positive correlation with the sample status (Figure 4E), whereas the turquoise gene module had the most significant negative correlation with the sample status (Figure 4F). Genes in these two modules were subsequently used for GO/KEGG enrichment analyses. The enrichment analysis results of blue module were shown in Figures 5A–D. With regard to BP, the blue module genes were significantly enriched in proteasomal protein catabolic process, neutrophil activation, and neutrophil mediated immunity (Figure 5A). As for CC, the blue module genes were mainly implicated in focal adhesion, cell–substrate adherens junction, and cell–substrate junction (Figure 5B). With respect to MF, the blue module genes were found in the pathway concerning electron transfer activity, oxidoreductase activity, and a multitude of other functions (Figure 5C). In addition, significant enrichment of the blue module genes in the KEGG pathway of Herpes simplex virus 1 infection was revealed (Figure 5D).

The enrichment analysis results of the turquoise module were as indicated in Figures 5E–H. With regard to BP, the turquoise module genes were significantly enriched in neutrophil activation, neutrophil mediated immunity, and neutrophil degranulation (Figure 5E). As for CC, the turquoise module genes were mainly implicated in cytoplasmic vesicle lumen, vesicle lumen, and secretory granule lumen (Figure 5F). With respect to MF, the turquoise module genes were those in the pathways of catalytic activity (acting on RNA), ubiquitin-like protein ligase binding, and ubiquitin protein ligase binding (Figure 5G). In addition, significant enrichment of the turquoise module genes in several KEGG pathways including Salmonella infection, NOD-like receptor signaling pathway, and NF-kappa B signaling pathway was revealed (Figure 5H).

### Network Analyses of Module Genes/Hub Genes

**Figure 6:**
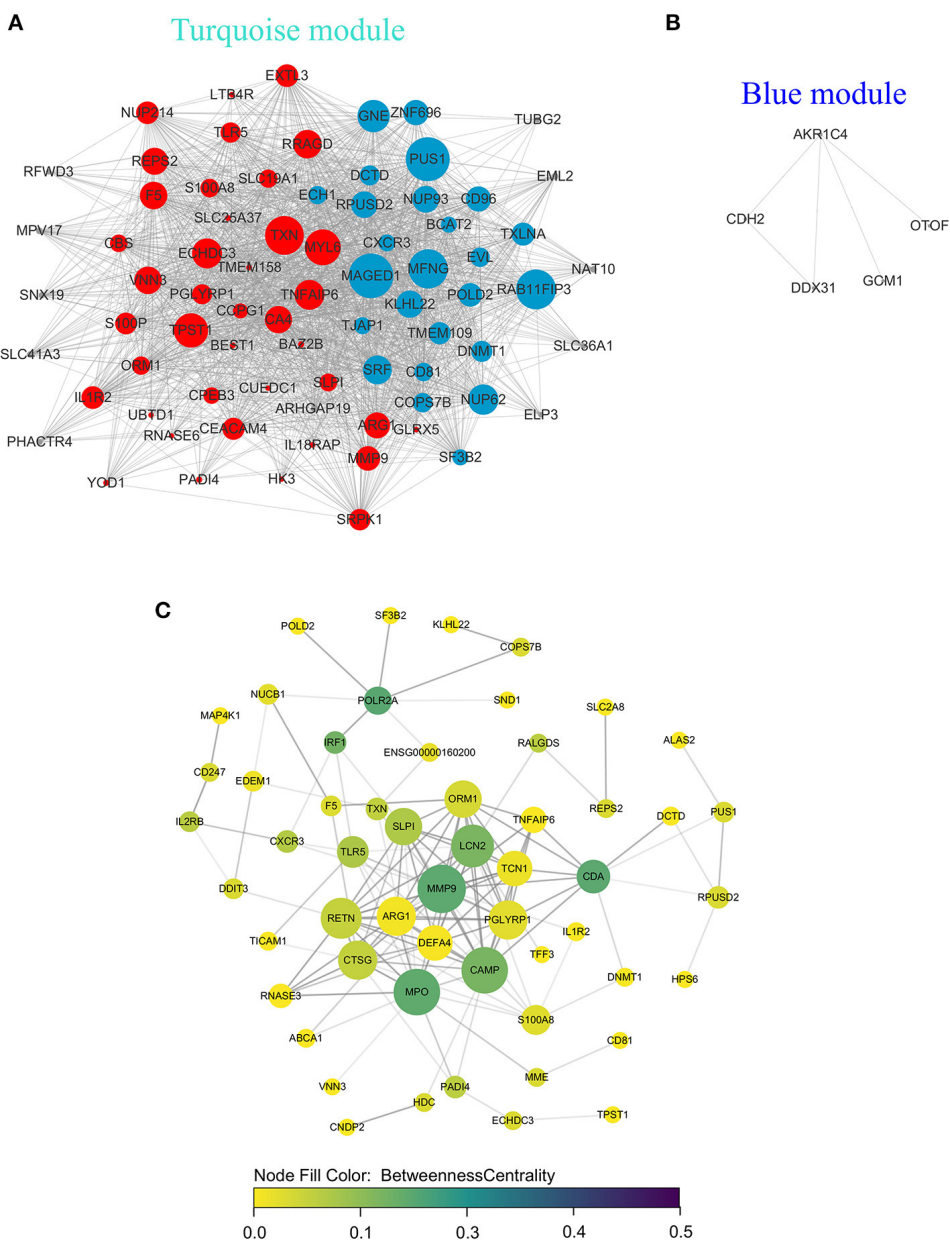
Network analyses of module genes/hub genes recognized by WGCNA at transcriptional and protein levels. In gene co-expression network of turquoise module **(A)**, blue module **(B)**, the red/blue circles represented up/downregulated module genes, respectively, and the gray circles represented module genes that were not differentially expressed; larger node size is associated with increased number of edges. **(C)** In protein–protein interaction network of hub genes, the node size (from small to large), node color (from yellow to green), and transparency of edges (from light to dark) were proportional to degree centrality (number of edges possessed by a node), betweenness centrality (the frequency that a node serves as a bridge along the shortest path between two other nodes), and combined score (evidence of interaction between two nodes based on different evidence channels, e.g., experimental data/association in curated databases), respectively.

**Figure 7:**
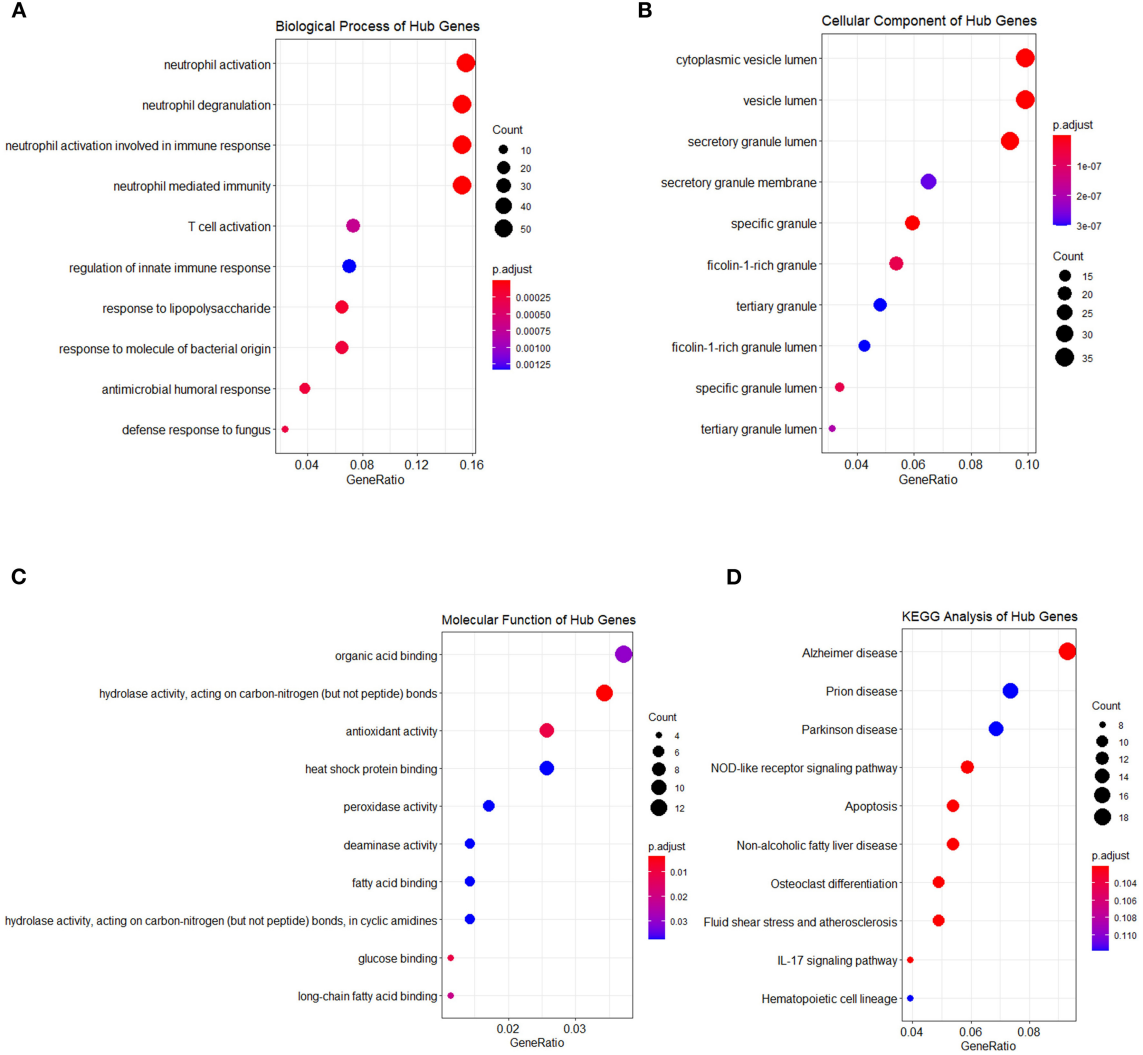
Functional enrichment analysis of hub genes. Hub genes were also subjected to enrichment analysis, categories are shown in **(A)** biological process, **(B)** cellular component, **(C)** molecular function, and **(D)** KEGG pathway, respectively. The dot size represented the count of differentially expressed genes, and the color depth represented the p-value-based significance.

Genes in the blue and turquoise modules were filtered based on node score and merged together, named module genes, and further visualized as a correlation network in Figure 6A, where up/downregulated genes (intersection of DEGs and genes in two modules) in the training set were, respectively, denoted by red/blue colors (nodes with larger size possess more edges). A total of two networks were generated, and all DEGs were clustered in the network based on the turquoise module (Figure 6A), whereas only five genes were filtered in the network based on the blue module (Figure 6B). Additionally, no correlation (edges) between turquoise network and blue network was observed due to the low inter-module correlation. Therefore, subsequent analyses were mainly focused on the turquoise module. Given that the correlation network at transcriptome level has been elucidated, we further analyzed the PPI network at protein level. Briefly, the intersection of module genes and DEGs identified above was defined as hub genes and searched against STRING online database to establish a PPI network; closely related proteins encoded by hub genes were shown in Figure 6C. The PPI network was subjected to topological analysis, and strong inter-connectivity was observed at the central part of the PPI network, including DEFA4, CTSG, ARG1, RETN, LCN2, PGLYRP1, MMP9, TNFAIP6, and TCN1, among which the intra-network importance of MMP9 and ARG1 was significantly increased (larger node size among hub genes) at protein level (Figure 6C, central area), compared with that at transcriptional level (relatively small node size among module genes, Figure 6A, bottom). Subsequently, these hub genes underwent GO/KEGG enrichment analyses. The enrichment analysis results of hub genes (Figures 7A–D) showed that with regard to BP, hub genes were significantly enriched in neutrophil activation, neutrophil degranulation, and neutrophil activation involved in immune response (Figure 7A). As for CC, hub genes were mainly implicated in cytoplasmic vesicle lumen, vesicle lumen, and secretory granule lumen (Figure 7B). With respect to MF, hub genes were found in the pathways of organic acid binding, hydrolase activity [acting on carbon–nitrogen (but not peptide) bonds], and antioxidant activity (Figure 7C). In addition, significant enrichment of hub genes in KEGG pathways associated with Alzheimer disease was revealed (Figure 7D).

### Validation of the Key Genes in the Test Set

**Figure 8:**
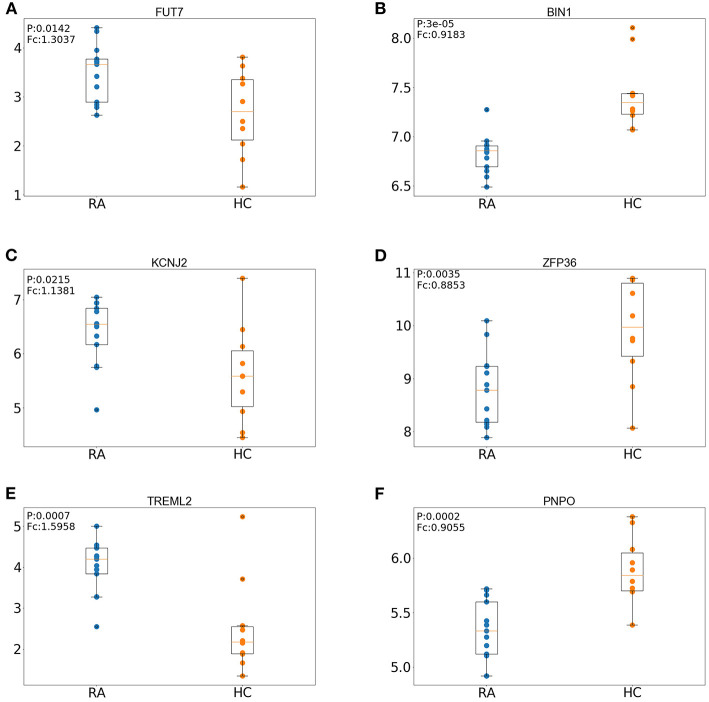
Expression patterns of the top three upregulated and three downregulated key genes in the RA (rheumatoid arthritis) and HC (healthy control) groups of the test set. **(A)** Expression patterns of FUT7. **(B)** Expression patterns of BIN1. **(C)** Expression patterns of KCNJ2. **(D)** Expression patterns of ZFP36. **(E)** Expression patterns of TREML2. **(F)** Expression patterns of PNPO.

We first defined a set of key genes as the intersection of hub genes (intersection of module genes and DEGs of the training set) and DEGs of the test set (Table 2). The expression pattern regarding the top three upregulated (FUT7, KCNJ2, TREML2) and downregulated (BIN1, ZFP36, PNPO) key genes was further verified in the test set (GSE55457, tissue samples) by comparing RA patients against controls. As shown in Figure 8, the box plot indicated that the expression pattern of these key genes resembled to that in the training set (blood sample): FUT7, KCNJ2, and TREML2 were significantly upregulated, accompanied by distinct downregulation of BIN1, ZFP36, and PNPO, suggesting the biological significance of these key genes across different samples and their potential to serve as biomarkers for RA.

**Table 2:** The information of 22 key genes.

**Symbols**	**logFC**	***p***	**Adj. *p***	**Threshold**
**FUT7**	0.9304	9.15e−18	8.58e−18	Up
**TREML2**	0.8808	9.16e−18	8.17e−17	Up
**KCNJ2**	0.9233	5.30e−17	3.53e−16	Up
**POLB**	0.7819	1.31e−15	6.45e−14	Up
AIM2	0.6428	2.62e−12	6.90e−11	Up
QPCT	0.6298	3.23e−9	5.41e−8	Up
PTP4A3	0.5064	3.44e−9	5.74e−8	Up
**TNFSF10**	0.5212	7.07e−8	9.91e−7	Up
**GLTSCR1**	−0.6983	5.07e−19	6.37e−18	Down
**ADM**	−1.1117	1.99e−16	1.04e−15	Down
**GPS1**	−0.7038	1.91e−16	1.01e−15	Down
**VEGFB**	−0.7276	8.69e−15	4.33e−15	Down
ECHDC2	−1.0442	1.33e−15	6.51e−14	Down
**TAF15**	−0.8912	1.34e−14	5.27e−13	Down
**SAFB**	−0.5298	1.69e−14	6.56e−13	Down
SNCA	−1.7333	7.64e−14	2.84e−14	Down
**BET1L**	−0.6322	1.31e−13	4.31e−12	Down
TOMM40	−0.5833	1.74e−13	5.74e−12	Down
LTBP4	−0.5541	2.87e−13	9.41e−12	Down
**PNPO**	−0.5628	5.19e−12	1.36e−12	Down
**BIN1**	−0.6311	5.74e−10	1.16e−10	Down
**ZFP36**	−0.8074	1.04e−10	2.01e−9	Down

*Key genes were obtained from intersection between DEGs in the test set and hub genes in the training set, including 8 upregulated genes and 14 downregulated genes, among which 15 features selected by Lasso (shown in bold) were used in subsequent machine learning modeling*.

### Immune Cell Infiltration Analysis

**Figure 9:**
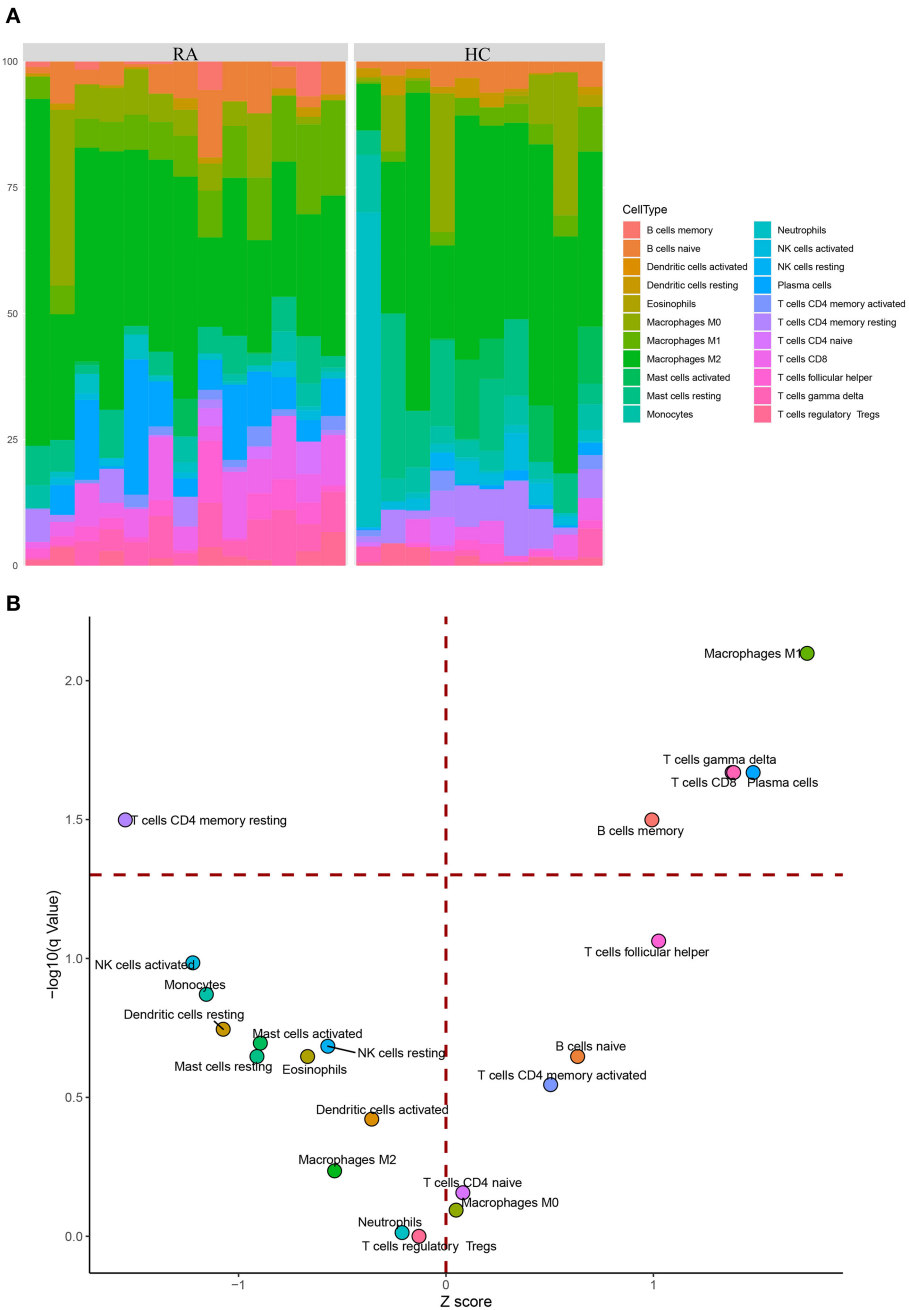
CIBER-SORT inference of immune cells composition in tissue samples. **(A)** Stacked barplot showing the percentages of 22 immune cell components of the RA and HC groups. **(B)** Volcano plot reveals distinct differences in relative compositions of immune cell populations between RA and HC samples. The y axis represents the –log10 transformation of q-value (adjusted p-value), and the red horizontal dash line indicates a q-value of 0.05.

After the analysis with CIBER-SORT, inferred immune cell compositions in the RA and HC groups were visualized (Figure 9A); the corresponding volcano plot (Figure 9B) showed that the percentages of five immune cells including macrophages M1, gamma delta T cells, CD8(+) T cells, plasma cells, and memory B cells were significantly elevated in the RA group (red horizontal dash line on the y axis indicates a q-value of 0.05). On the contrary, the proportion of resting CD4(+) memory T cells was reduced in the RA group, in distinct contrast to the elevated level of activated CD4(+) memory T cells.

### Machine Learning Model Validation

**Figure 10:**
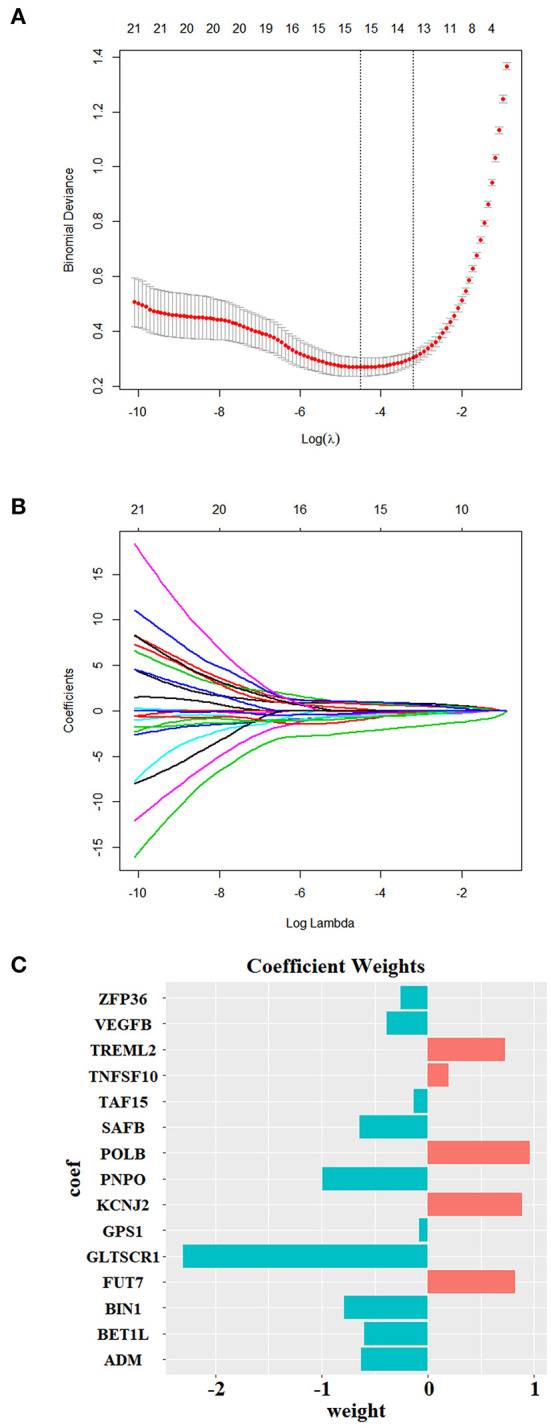
Feature selection using Lasso. **(A,B)** The LASSO penalized model showed that when Log λ = −4.51, the minimum binomial deviance for the selected λ was ~0.3, where the coefficient of 15 selected features (with non-zero coefficients) was calculated and shown in **(C)**.

**Figure 11:**
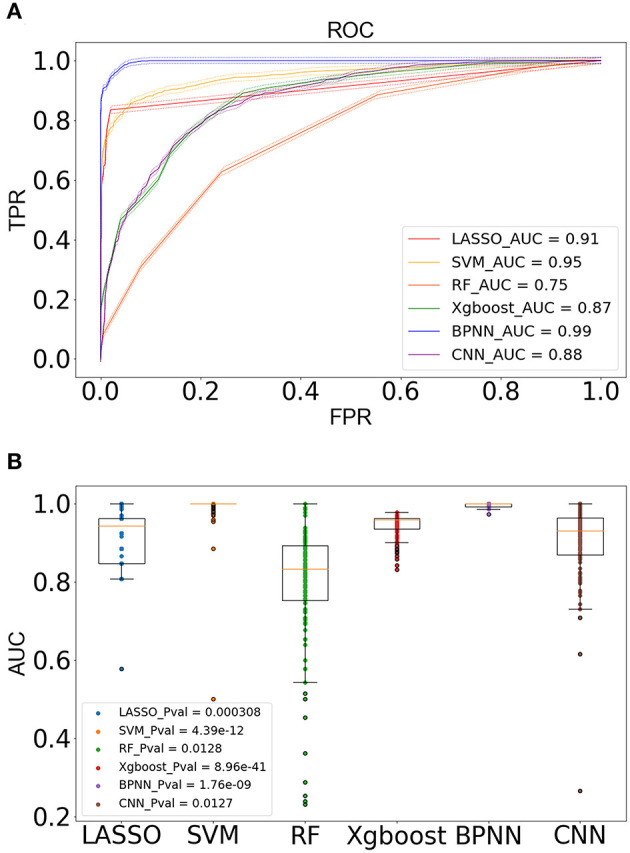
Modeling on independent test set for verification. **(A)** The ROC diagram showed the prediction effect of different models on the test set, with 95% confidence interval being marked with dotted lines. The AUC value of the model represents the area under the ROC curve. The closer the AUC to 1, the better the generalization of the model. **(B)** The box plot of the AUC value showed the distribution of 1,000 AUC values obtained by the model in 1,000 independent random verifications. The narrower the box, the more stable the prediction effect of the model.

Our machine learning methods were based on pre-defined key genes. First, LASSO algorithm was used for selection of pivotal key genes that were appropriate for subsequent machine learning validation; the λ value corresponding to the smallest binomial deviance was selected as the penalty coefficient, whereby 15 features were obtained for building the predictive model (Figures 10A,B). The weights of the gene features were shown in Figure 10C. Next, the evaluation of up to six different machine learning classification models (“LASSO,” “SVM,” “RF,” “Xgboost,” “BPNN,” “CNN”) was carried out. The performance of the six models was demonstrated using ROC, which was plotted with TPR (sensitivity) against FPR (1 – specificity) (Figure 11A). ROC curve for a specific machine learning method is generated by varying thresholds that result in trade-off between sensitivity and specificity. Therefore, AUC can be used to evaluate the performance of a specific model; the higher the AUC, the better the effectiveness of prediction. By comparing the AUC of the six machine learning models, BPNN (0.99) appears to perform the best among the six machine learning models; LASSO (0.91) and SVM (0.95) also did well in this regard. Notably, aside from the RF model with AUC lower than 0.8, the other models achieved relatively high effectiveness (AUC >0.85) in RA prediction. These observations were consistent with the box plot that showed the distribution of 1,000 AUC values obtained by six machine learning models in 1,000 independent random verifications (Figure 11B). As shown in Supplementary Figure 1, during 1,000 iterations in the training process of “BPNN,” “CNN” using batch gradient descent method, the fitting effect increased with the number of iteration (up to 1,000 iterations), which was reflected in lower loss value. The detailed information of this section was shown in Table 3 (test set) and Supplementary Table 3 (training set), respectively.

**Table 3:** The evaluation indicators of the six models were extracted from the test results of independent datasets, and the value of each indicator was obtained through 1,000 independent random verifications.

**Model**	**Cut-off**	**Sensitivity**	**Specificity**	**PPV**	**NPV**	**Accuracy**	**AUC (CI: 95%)**	***p***
LASSO	0.4	53.1% ± 0.0034	99.7% ± 0.0003	99.8% ± 0.0003	63.5% ± 0.0020	73.9%	0.91 ± 0.0013	3.08e−04
	0.5	50.5% ± 0.0235	99.8% ± 0.0003	99.8% ± 0.0002	62.2% ± 0.0019	73.9%		
	0.6	46.0% ± 0.0038	99.9% ± 0.0002	98.9% ± 0.0020	60.2% ± 0.0020	73.9%		
SVM	0.4	92.3% ± 0.0033	85.8% ± 0.0037	91.4% ± 0.0019	92.2% ± 0.0030	87.0%	0.95 ± 0.0010	4.39e−12
	0.5	90.5% ± 0.0035	89.7% ± 0.0032	93.7% ± 0.0017	91.7% ± 0.0025	91.3%		
	0.6	88.3% ± 0.0038	93.2% ± 0.0028	95.8% ± 0.0015	89.8% ± 0.0028	95.7%		
RF	0.4	83.5% ± 0.0049	50.4% ± 0.0065	71.9% ± 0.0031	69.3% ± 0.0065	56.5%	0.75 ± 0.0032	1.28e−02
	0.5	59.3% ± 0.0068	80.5% ± 0.0050	80.2% ± 0.0049	65.4% ± 0.0044	73.9%		
	0.6	59.3% ± 0.0068	80.5% ± 0.0050	80.2% ± 0.0049	48.9% ± 0.0066	60.9%		
Xgboost	0.4	98.7% ± 0.0006	24.4% ± 0.0040	63.6% ± 0.0012	68.2% ± 0.0086	73.9%	0.87 ± 0.0006	8.96e−41
	0.5	79.9% ± 0.0052	77.3% ± 0.0028	82.9% ± 0.0024	80.9% ± 0.0029	82.6%		
	0.6	9.85% ± 0.0026	99.9% ± 0.0002	51.9% ± 0.0098	46.4% ± 0.0010	52.2%		
BPNN	0.4	98.7% ± 0.006	95.1% ± 0.0010	96.5% ± 0.0007	98.4% ± 0.0007	95.7%	0.99 ± 0.0000	1.76e−09
	0.5	95.3% ± 0.0007	97.3% ± 0.0009	98.0% ± 0.0006	94.4% ± 0.0009	95.7%		
	0.6	92.7% ± 0.0006	98.4% ± 0.0007	98.8% ± 0.0005	91.3% ± 0.0007	95.7%		
CNN	0.4	81.5% ± 0.0037	78.1% ± 0.0043	85.0% ± 0.0024	78.5% ± 0.0039	87.0%	0.88 ± 0.0019	1.27e−02
	0.5	72.4% ± 0.0045	84.8% ± 0.0036	87.2% ± 0.0028	74.1% ± 0.0031	82.6%		
	0.6	61.7% ± 0.0050	90.0% ± 0.0027	90.0% ± 0.0029	68.2% ± 0.0031	82.6%		

## Discussion

In this study, we first identified numerous DEGs (between RA patients and controls) in the training set/test set. Gene modules (blue/turquoise modules) strongly associated with RA in the training set were subsequently discovered by WGCNA, whose intersection with DEGs in the training set was defined as hub genes. Network analyses revealed the correlation among hub genes at both transcriptome (WGCNA node score) and protein (PPI network combined score) levels. Afterward, the intersection between DEGs in the training set and hub genes was defined as key genes and underwent selection by LASSO algorithm before being used as features in machine learning model validation. Finally, the biological significance of these features was supported by relatively high AUC (>0.85) achieved by 5 out of 6 machine learning models.

In 11 GEO datasets based on blood samples (training set), we discovered a total of 451 DEGs that were significant between RA patients and controls, among which 231 upregulated and 220 downregulated genes were screened using a stringent threshold. These DEGs underwent subsequent GO term enrichment and KEGG pathway analysis, through which we further discovered several pathways where DEGs were significantly enriched. As for BP, DEGs were related to neutrophil associated pathways. Neutrophils play a pivotal role in the initiation of RA; these cells will be activated after migrating into the articular cavity where they exert regulatory functions, such as generating cytokines that affect other immune cells, thereby sustaining the inflammation status and contributing to the pathogenesis of RA (Chen et al., 2018b). With regard to CC, DEGs were mainly associated with lumen, such as cytoplasmic vesicle lumen and secretory granule lumen, indicating that RA might affect the lumen biogenesis, which was not previously reported. Enrichment of DEGs in the MF pathway displayed a greater diversity, which involved organic acid binding, antioxidant activity, and hydrolase activity acting on non-peptide carbon–nitrogen bonds, among which the potential of antioxidant as an alternative RA therapy has been proposed according to the evaluation of plasma oxidant/antioxidant status in RA (Jaswal et al., 2003). KEGG pathway analysis of DEGs demonstrated relatively high enrichment in the pathway concerning Alzheimer disease; although the enrichment in Parkinson disease was less significant, these pathways were strongly correlated with neurodegenerations. Their association with RA was supported by several publications; specifically, a Mendelian randomization study proposed a correlation between RA and Alzheimer disease (Bae and Lee, 2019); co-methylation relation study revealed that samples of RA and Parkinson disease shared 337 significantly altered (vs. their respective control) methylation gene pairs (Tang et al., 2018).

To explore the way through which these DEGs worked in concert to affect the pathogenesis of RA, we utilized WGCNA to select highly co-expressed gene modules for further investigation. As a result, a total of five modules were defined, among which two modules (blue/turquoise) exhibited the most significant positive/negative correlation with the sample status; therefore, these two modules were merged together as module genes for in-depth study. Pathway enrichment analysis of these modules showed that: 1, consistent with the results of DEGs, both modules were mainly enriched in neutrophil associated process with regard to BP category; 2, for CC, genes in the blue module were predominantly implicated in adhesion and junction, whereas genes in the turquoise module were clustered in lumen, agreeing with the result of DEGs; 3, in terms of MF, genes in the blue module were responsible for NADH dehydrogenase activities, whereas genes in the turquoise module participated in various molecular binding activities, especially ubiquitin protein ligase binding; 4, similar to DEGs, genes in the blue module were mainly associated with neurodegeneration-associated KEGG pathways, whereas genes in the turquoise module were connected with immune-associated KEGG pathways [nuclear factor (NF-kappa) B signaling pathway/T cell receptor signaling pathway]. Collectively, pathways in which genes in the blue module were enriched (adhesion/junction, NADH dehydrogenase activities, and neurodegeneration) were positively correlated with RA, whereas their turquoise counterparts (lumen, ubiquitin binding, and immunity) were quite the opposite. Some supporting evidence was discussed below: the expression of several adhesion molecules was previously discovered in rheumatoid synovium, and these molecules promote the pathogenesis of RA through regulating synovial production (Haskard, 1995; Liao and Haynes, 1995); the mutations of the A20 [tumor necrosis factor (TNF) inducible protein 3] at ZnF7 (zinc finger 7) ubiquitin binding domain lead to arthritis in the mouse model (Polykratis et al., 2019). Through visualization with correlation network, strong intra-connectivity was found within modules. The intersection of module genes and previously identified DEGs was named hub genes, and the correlation among hub genes was further visualized through network analyses. We found that MMP9 and ARG1 were more pivotal at protein level (among hub genes) than at transcriptional level (among module genes), as these proteins exhibited increased level of degree centrality and betweenness centrality. Additionally, *MMP9* and *ARG1* were upregulated DEGs identified in our training set, which was supported by previous reports. Indeed, MMP9 is distinctly increased in serum, and especially in the synovial fluid of RA patients, partially through conferring synovial fibroblast with survival benefits, thereby inducing inflammation and degradation of the cartilage (Xue et al., 2014). Moreover, ARG1 was reported to be strongly associated with polyamine and nitric oxide (NOS) in RA, and high ARG1 activity is considered as a frequent feature for RA patients (Panfili et al., 2020). In contrast, pivotal genes, such as *MAGED1, RAB11FIP3*, and *PUS1*, that were correlated with the vast majority of the turquoise module genes were less prominent at protein level.

Enrichment analyses were performed to interpret the biological significance of the newly defined set of genes; the majority of results concerning BP, CC, and KEGG pathway analysis were highly consistent with that of DEGs or blue/turquoise modules, except for enrichment in GO MF terms that varied across a wide range of MFs, including binding functions (organic acid binding, heat shock protein binding, fatty acid binding, glucose binding, etc.) and regulation of enzyme activity (hydrolase, antioxidant, peroxidase, and deaminase activities). Some of these functions were also related to arthritis: research on pre-RA subjects and matched controls in a cohort study showed that erythrocyte membrane levels of the n−6 polyunsaturated fatty acids—linoleic acid (PUFA LA) were inversely associated with RA development (de Pablo et al., 2018); the occurrence of abnormal glucose metabolism in RA patients was significantly higher (Pi et al., 2017). A cross-sectional study demonstrated that antioxidant levels were elevated in RA patients by comparing controls; worse still, these patients were unable to avoid impairments induced by oxidation (Garcia-Gonzalez et al., 2015).

To further explore biologically significant candidates, the key genes were defined as the intersection of hub genes (intersection of module genes and DEGs of the training set) and DEGs of the test set. However, *MMP9* and *ARG1* that played a central role in the PPI network were not among the key genes, and we hypothesized that these genes might play a major role on peripheral blood instead of RA tissue. Nevertheless, we verified the expression patterns of the key genes in tissue samples of RA patients by comparing with controls and discovered the top three upregulated (*FUT7, KCNJ2, TREML2*) and downregulated (*BIN1, ZFP36, PNPO*) key genes. As expected, these key genes exhibited similar expression patterns in tissue samples; we discuss their potential roles in RA based on our current results in the following lines. *FUT7* gene encodes fucosyltransferase-VII, an important mediator for synthesizing selectin ligands (Sarraj et al., 2014) and leukocyte adhesion (Wang et al., 2013). Mice with mutated *FUT4* and *FUT7* exhibited remarkable deficiencies in terms of leukocyte recruitment at the presence of acute inflammation (Homeister et al., 2001). Importantly, upregulated *FUT7* was reported in synovial tissues of RA, and its potential correlation with M1 inflammatory macrophages was also verified by quantitative reverse transcription PCR (qPCR) (Li et al., 2014). K_ir_2.1, also called inward-rectifier potassium ion channel, is a lipid-gated ion channel encoded by the *KCNJ2* gene and a mediator of inward-rectifier K^+^ current (IK1) (Li et al., 2016), and the potential association between K_ir_2 and immune response has been described, but yet it remains elusive. For instance, interferon gamma (IFN-*γ*) is secreted by Th1 (CD4+) cells, and the inverse correlation between IFN-*γ* and IK1/K_ir_2.1 expression was found in the rat heart with myocardial infarction, where Th1 cells responded to the stimulation by elevating the level of IFN-*γ*; the regulatory role of CD4 cells on IK1/K_ir_2.1 expression was therefore ascribed to IFN-*γ* (Li et al., 2016). *TREML2*, also termed as myeloid cells (TREM)-like transcript 2 (*TLT-2*), is stably expressed by CD8(+) T cells, when binding to T cell proliferation co-stimulator *B7-H3* (*CD276*) (Chapoval et al., 2001), T cell proliferation will be promoted; the regulatory role of *TLT-2* was therefore attributed to activated *TLT-2*–*B7-H3* signaling cascade (Hashiguchi et al., 2008). The pro-inflammatory nature of *TREML2* was also indicated in a previous study showing an upregulation of *TREML2* in response to inflammation (King et al., 2006). Bridging Integrator-1 is encoded by the human *BIN1* gene (Negorev et al., 1996) and predominantly expressed in the brain and muscle (Wechsler-Reya et al., 1997), which is a multifunctional protein that also serves as a tumor suppressor through interacting with Myc; hence, Myc box-dependent-interacting protein is an alias for the *BIN1* gene (Sakamuro et al., 1996). Moreover, increasing bodies of evidence suggested that altered *BIN1* might affect the common late onset of AD (LOAD) through the tau pathology pathway (Tan et al., 2013). Intriguingly, the potential inflammatory role of *BIN1* was reported in the aging *BIN1* knockout mice model that exhibited an elevated incidence of inflammation (Chang et al., 2007). The *ZFP36* gene (also known as zinc finger protein 36 homolog) encodes tristetraprolin (*TTP*), a negative regulator of many pro-inflammatory genes, including TNF-α that participated in the pathogenesis of RA and other inflammatory diseases (Feldmann, 1996). Knockout of *ZFP36* was shown to provoke severe erosive arthritis in mice (morphologically resembles human RA) (Taylor et al., 1996), such inflammatory response was attributed to the inhibitory effect of *TTP* on *TNF* (Carrick et al., 2006). The pyridoxine-5′-phosphate oxidase (PNPO) enzyme plays a pivotal role in pyridoxine conversion (Jaeger et al., 2016) and the synthesis of activated vitamin B6 [pyridoxal 5′-phosphate (PLP)], which is also involved in a board spectrum of BPs including metabolizing amino acids and synthesizing nucleic acids (Khayat et al., 2008). Up to now, relatively few studies have been focusing on the correlation between *PNPO* and disease pathogenesis, one of which proposed that infantile seizure was a consequence of *PNPO* deficiency (Khayat et al., 2008).

Among other key genes, *TNFSF10* and *VEGFB*, respectively, belong to two established RA-associated protein families, namely, TNF superfamily and vascular endothelial growth factor (VEGF) family. TNFSF superfamily is involved in the simulation of several immune cells (including T and B lymphocytes) and therefore considered a hallmark of autoimmune diseases (Croft et al., 2012), among which TNF-α is enriched in the synovial fluid of RA patients that exerts pro-inflammatory effects and regarded as a therapeutic target of RA (Radner and Aletaha, 2015). VEGF, as an angiogenic factor that occurs in response to impairments, could be elevated by cytokines that promote inflammation, and the serum level of VEGF in RA patients was proposed as an indicator of RA progression (Taylor, 2002). Combined with the current results, *TNFSF10* and *VEGFB* might also participate in the pathogenesis of RA through similar ways and serve as RA markers.

As an autoimmune disease, the onset of RA is accompanied by a series of immune processes, wherein immune cells play a fundamental role. To gain a deeper understanding of the key genes, we sought to link the expression patterns of the key genes with immune cells by analyzing the immune cell composition in tissue samples. The promoted local recruitments of five types of immune cells in RA tissues were inferred by CIBER-SORT, among which increased proportions of CD8(+) T cells and M1 inflammatory macrophages were consistent with the verification regarding 2 out of 3 top upregulated genes (elevated *TREML2* and *FUT7* corresponded to increased proportions of CD8(+) T cells and M1 inflammatory macrophages, respectively) in RA tissue samples. Intriguingly, in RA tissues, the percentage of resting CD4(+) memory T cells in RA was significantly reduced by comparing the elevated proportion of activated CD4(+) memory T cells, such trade-off suggested that resting CD4(+) memory T cells might be activated in response to the inflammatory stimulation of RA. These results indicated that the change in RA immune microenvironment might be associated with our currently identified key genes. Collectively, among the six top key genes, to our knowledge, *FUT7* and *ZFP36* were previously reported to be related to RA, whereas *TREML2, KCNJ2*, and *BIN1* might be associated with inflammatory response. The one remaining gene *PNPO* is a novel potential RA biomarker predicted in the current study. The elevated *TREML2* and *FUT7* expression was consistent with the inference of CIBER-SORT immune cell alteration.

Considering that the expression of the top key genes was validated to be consistent across different (blood/tissue) RA patient samples, we used the LASSO penalized model to further select pivotal key genes that were appropriate for subsequent validation. A total of 15 pivotal key genes were selected, and, as expected, the top three up/downregulated (FUT7, KCNJ2, TREML2/BIN1, ZFP36, PNPO) key genes were also incorporated. These biomarkers were used as features to train multiple machine learning algorithms to validate their biological significance. Previously, WGCNA and machine learning were used to uncover causative factors in RA; for instance, Ma et al. (2017) used two datasets (GSE55235 and GSE77298) that contained a total of 26 RA samples and 17 HCs to unearth core players in RA through WGCNA. Although the key genes described in their study did not overlap with that identified in our current study, it is notable that *TNFSF10* (the key gene identified in the current study) was also among the top 10 characteristic genes (without heterogeneity between two datasets) reported by Xing et al. (2011); Ma et al. (2017) used Bayesian network in conjunction with Monte Carlo simulation to predict genes with novel roles (which was not directly associated with RA therapeutic target, TNF-α) in RA treatment, whereby dozens of genes were predicted to play novel roles in RA by impacting Disease Activity Score 28 (DAS28) or joint health. Platzer et al. (2019) adopted machine learning to generate gene expression-based models for distinguishing between HC/RA and early RA/other related arthritis (e.g., arthralgia) based on several DEGs identified by single-variable comparisons. By comparing these machine learning-based reports, we did not find intersection between our currently identified key genes and those identified in the abovementioned machine learning-based reports, which might be attributed to different research goals and the scales of datasets.

Likewise, studies of machine learning had attained successes in the prediction of RA, for instance, histologic data were used to establish machine learning model for stratifying synovial subtypes of RA (Orange et al., 2018); multiple features of RA and non-RA categorization including texture and shape were integrated for RA classification (Bardhan and Bhowmik, 2019).

However, relatively limited studies were focused on the potential of machine learning as an evaluator of biological significance. In our present study, pivotal biomarkers that were selected by LASSO (15 pivotal key genes) were validated by machine learning model validation, and the results showed that aside from “RF,” AUC of multiple machine learning methods (“LASSO,” “SVM,” “Xgboost,” “BPNN,” “CNN”) exceeded 0.85, especially BPNN (with an AUC of 0.99), which was higher (although not directly comparable) than the AUC achieved by the aforementioned reports. As suggested in previous review, eliminating redundant features and focusing on fewer elements that are relevant to the phenotypes could improve the performance of a classifier, as empirical knowledge was incorporated into the procedure (Libbrecht and Noble, 2015). Therefore, the achievement of the current machine learning models might be ascribed to the pivotal key genes that served as features.

Although we have achieved significant results in our current study, several limitations should be noted. First, analyses were mainly performed based on transcriptome information (although a PPI network was constructed and analyzed); therefore, posttranslational modulations that play pivotal roles in the pathogenesis of autoimmune diseases including RA (Opdenakker et al., 2016) were not put into consideration for most part of the study. Second, we did not find well-established RA markers, such as C-reactive protein (CRP) (Choy, 2012), interleukin (IL)-6 (Srirangan and Choy, 2010), or TNF-α (Saklatvala, 1986) (although another member of TNF superfamily, TNFSF10, was discovered as a key gene in the present study), which might be ascribed to the integration process in the current study, during which massive datasets were aggregated to obtain the training set, at the cost of excluding some important genes with incomplete information (with ID that was not shared by all datasets or with proportion of missing value >50% across all datasets).

In summary, the present study discovered several enriched pathways in RA patients based on DEGs, and the corresponding hub genes were determined by WGCNA and visualized using the PPI network. By incorporating the genetic information from the test set, 22 key genes were subsequently defined, among which *FUT7* (Li et al., 2014) and *ZFP36* (Taylor et al., 1996) were reported to be involved in the pathogenesis of RA; *TNFSF10* (Croft et al., 2012) and *VEGFB* (Taylor, 2002) belonged to known RA-associated families; *TREML2* (Chapoval et al., 2001), *KCNJ2* (Li et al., 2016), and *BIN1* (Chang et al., 2007) were proposed to be associated with immune response; *TREML2* and *FUT7* exhibited expression patterns that were consistent with our current inferential results of immune cells composition, whereas *PNPO* is not yet reported in RA and deserves further investigation. The preceding key genes were among the 15 pivotal features selected by LASSO, and their biological significance was further confirmed through machine learning model validation. Taken together, our currently identified key genes might provide novel perspective in understanding RA pathogenesis or serving as biomarker for RA diagnosis. Their correlation with immune response/immune cells also confers them the potential as therapeutic target of RA, since the suppression of harmful properties in immune cells has been proposed as a feasible approach to alleviate RA through preventing inflammation (Xue et al., 2019).

## Data Availability Statement

The original contributions presented in the study are included in the article/Supplementary Material, further inquiries can be directed to the corresponding author/s.

## Author Contributions

JX and RW wrote the manuscript and interpreted the data. XC contributed to the analysis of data and drafted the manuscript. ZY designed the study and revised the manuscript. All the authors have read and approved the final version of the submitted manuscript.

## Conflict of Interest

The authors declare that the research was conducted in the absence of any commercial or financial relationships that could be construed as a potential conflict of interest.

## Supplementary Material

Supplementary Figure 1 The trend of loss value with the number of iterations. In 1,000 iterations with batch gradient descent method, the loss value was significantly lower with the increase in the number of iteration, indicating that the fitting effect increase with iteration.
