# Supplementary figures and images for: Coupling of Co-expression Network Analysis and Machine Learning Validation Unearthed Potential Key Genes Involved in Rheumatoid Arthritis
Source: Front Genet. 2021 Feb 11;12:604714. doi: 10.3389/fgene.2021.604714 (PMC7905311; doi:10.3389/fgene.2021.604714)

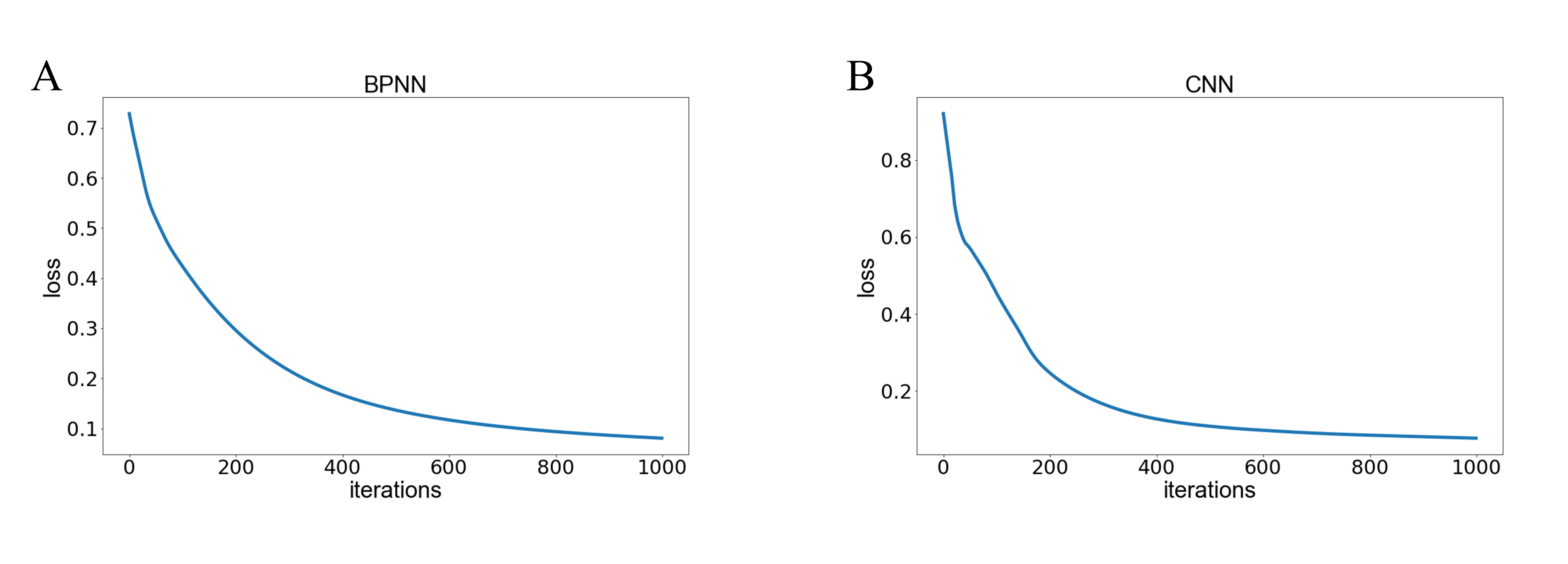

Supplement: Supplementary Figure 1 — The trend of loss value with the number of iterations. In 1,000 iterations with batch gradient descent method, the loss value was significantly lower with the increase in the number of iteration, indicating that the fitting effect increase with iteration. [file Image_1.PNG]
